# The landscape of actionable genomic alterations in lung adenocarcinomas in India

**DOI:** 10.3389/fgene.2023.1256756

**Published:** 2023-12-13

**Authors:** Rakesh Sharma, Aruna Priya Kamireddy, Syed Meera Hussaini, Soma Chatterjee, Qurratulain Hasan, Jugnu Jain

**Affiliations:** ^1^ Sapien Biosciences Private Limited, Hyderabad, Telangana, India; ^2^ Department of Genetics and Molecular Medicine, Kamineni Hospitals, Hyderabad, Telangana, India

**Keywords:** next-generation sequencing, non-small cell lung cancer, precision medicine, molecular landscape, biomarker, actionable alteration, targeted therapy

## Abstract

Lung adenocarcinoma (LUAD), the most prevalent form of non-small cell lung cancer (NSCLC), remains a leading cause of cancer-related death globally, including in India, with a 5-year survival rate below 10%. Despite these grim statistics, recent advances in the use of next-generation sequencing (NGS) for identifying genetic alterations and the emergence of targeted therapies have opened new possibilities for personalized treatment based on distinct molecular signatures. To understand the molecular pattern of NSCLC, a retrospective study was conducted with 53 Indian LUAD patient samples, using a targeted NGS panel of 46 cancer-relevant oncogenes to identify clinically relevant variants. Pathogenic or likely pathogenic variants were detected in 94% of the 53 cases. Non-synonymous mutations, rearrangements, copy number alterations, insertions, and deletions of functional relevance were observed in 31 out of 46 genes. The most frequently mutated genes included *TP53* (52.8%) and *EGFR* (50.9%), followed by *RET*, *PIK3CA* and *ERBB2*; some patients had multiple alterations in the same gene. Gender-based enrichment analysis indicated that *ALK* and *IDH2* alterations were more prevalent in females, while *TP53* and *PTEN* were more common in males. No significant correlation was found between mutations and other clinicopathological attributes, such as age, stage, and subtype. A higher prevalence of *EGFR*, *RET*, *PIK3CA*, *ERBB2* and *ALK* mutations were observed compared to previous LUAD genetic studies coupled with a lower frequency of *KRAS* mutations. Clinically actionable variants were annotated using OncoKB and categorized into the four therapeutic levels based on their clinical evidence. Seventy-nine percent of cases had at least one clinically actionable alteration. Most patients (39.6%) had the highest level of actionability (Level 1) wherein an FDA-approved drug is available specifically for the observed mutation in lung cancer patients. *EGFR* Exon19 in-frame deletions and *EGFR* L858R were the most frequent among targetable variants (20.7%). These findings emphasize the importance of a selective NGS panel in enabling personalized medicine approaches by identifying actionable molecular alterations and informing the choice of targeted therapy for more effective treatment options in Indian NSCLC patients.

## 1 Introduction

Lung cancer remains one of the leading causes of cancer-related deaths globally, with an overall 5-year survival rate ranging from 4%–17% in the United States and 10% in India (Hirsch et al., 2017; Hawkes, 2019; Mathur et al., 2020). Despite these grim statistics, advancements in next-generation sequencing (NGS) technology have led to a deeper understanding of the underlying biology and molecular mechanisms driving the progression of lung cancer (Hensing et al., 2014). NGS has enabled the identification of targetable driver mutations, mechanisms of resistance, quantification of tumor mutational burden, microsatellite instability and germline mutations (Erika Ruiz-Garcia & Horacio Astudillo-de la Vega, 2018; Howlader et al., 2020; Wang et al., 2021). NGS has also proven valuable in correlating clinicopathological characteristics, genomic profile, grade, and tumor recurrence with these genetic variants (Caso et al., 2020). The integration of such analyses has paved the way for stratification of patients into specific subgroups, and the use of FDA-approved targeted therapies and immunotherapies matched with specific driver mutations identified in individual patients (Hensing et al., 2014; Vargas and Harris, 2016).

The NCCN Clinical Practice Guidelines in Oncology for non-small cell lung cancer (NSCLC), recommend the molecular testing of clinically proven predictive biomarkers, namely, *ALK*, *BRAF*, *EGFR*, *KRAS*, *MET* exon14 skipping, *NTRK1/2/3*, *RET* and *ROS1* genes, and a few emerging ones such as *MET* amplification and *ERBB2* mutations (Kristina Gregory et al., 2022). Additional targeted drugs for other genes are available but not approved for NSCLC as of now. With NGS profiling identifying more actionable mutations in NSCLCs, these drugs may be considered for expansion for the treatment of NSCLC patients. Further, since resistance to targeted therapies often develops due to the cancer cells proliferating and mutating rapidly, NGS can also help identify validated biomarkers predictive of resistance to targeted drugs.

Genetic data of Indian lung cancer patients is under-represented in publicly available databases. Our study aims to address this gap by determining the molecular landscape of 53 Indian NSCLC patient samples that were retrospectively collected, using an NGS hotspot panel. Our study identified several pathogenic and likely pathogenic alterations in most patient samples, including several potentially actionable variants with known therapeutic implications and other emerging potential therapeutic targets in NSCLC. These findings underscore the utility of such targeted NGS panels in detecting the prevalent mutations and leveraging their biological and clinical implications for Indian NSCLC patients to benefit from targeted therapy options.

## 2 Materials and methods

### 2.1 Ethical approval for the study

Formalin-fixed paraffin-embedded (FFPE) blocks of lung cancer cases obtained by the biobank with appropriate waiver of consent approvals from the institutional ethics committee (IECs) of Apollo hospitals were used in this study. The use of the biobanked FFPE samples and associated diagnostic data for NGS analysis was further approved by the IEC of the biobank, constituted as per the Indian Council of Medical Research (ICMR) 2017 and DHR guidelines (Protocol SBS-IEC-2020-05 titled “Utilization of retrospective FFPE tissue blocks for research and development at Thermo Fisher Scientific” and Protocol SBS-IEC-2022-01 titled “Analysis of sequencing data of biobanked cancer tissue samples for translational research and publication”). FFPE samples and data were coded by the biobank to protect patient confidentiality and privacy as per ICMR guidelines.

### 2.2 Lung cancer FFPE blocks and data

A total of 103 lung cancer resection cases drawn from the years 2004–2021 were profiled. Demographic data such as age and gender of the patient, and diagnostic data for the surgical samples were retrieved from the biobank’s curated medical records. A haematoxylin and eosin (H&E) stain of each block was used to re-confirm their quality and histological diagnosis by a clinical pathologist in order to remove any risk of inaccuracies stemming from the use of archived blocks and records.

### 2.3 Next-Generation Sequencing

Sections of 20-microns were used from FFPE blocks and sequenced by ThermoFisher Scientific (TFS) using their proprietary Oncomine™ Dx Express Test (ODxET™) on their Ion Torrent™ Genexus™ Integrated Sequencer. TFS performed the raw data processing, variant calling, and annotation, and provided a comprehensive list of annotated variants based on their gene class (gain-of-function GOF, or loss-of-function LOF) and variant class annotations (Supplementary data).

### 2.4 Data analysis

The list of genetic variants generated by TFS was further filtered to remove synonymous mutations that lacked variant identifiers such as COSMIC or arbitrarily assigned identifiers. Three LUAD cases (*n* = 3) were excluded from the study due to “poor” DNA or RNA QC scores. The specifics for calculating composite DNA and RNA QC scores are outlined in Supplementary methods.

MutationMapper and OncoPrinter from the cBioPortal Cancer Genomics (Cerami et al., 2012; Gao et al., 2013) were used for data visualization purposes. Additional annotations and drug-alteration matching were performed using OncoKB (Chakravarty et al., 2017). All statistical analyses were performed using the GraphPad Prism 6 software (GraphPad software Inc., La Jolla, CA, United States).

## 3 Results

### 3.1 Clinical and histopathological characteristics

In this study, 53 cases of primary non-metastatic LUAD were selected from the 103 lung cancer cases that were profiled using a 46 gene hotspot Oncomine™ Dx express test. These 53 cases included 2 cases that had NACT prior to surgery and 2 that had locally relapsed within 6 months. Among the 53 cases, 29 (54.7%) were classified as adenocarcinoma with features not otherwise specified, while the remaining cases were categorized into various subtypes according to the 2021 WHO classification of lung tumors (Table 1).

**TABLE 1:** Histopathological classification (*n* = 53).

WHO 2021 classification		No. of cases (%)
Adenocarcinoma (*n* = 49)		
	NOS	29 (54.7%)
	Bronchioloalveolar carcinoma	6 (11.3%)
	Invasive	4 (7.5%)
	Mucinous	5 (9.4%)
	Papillary	2 (3.8%)
	Clear cell	2 (3.8%)
	Acinar	1 (1.9%)
Adenosquamous carcinoma		3 (5.7%)
Large cell neuroendocrine carcinoma with areas of adenocarcinoma		1 (1.9%)

The patient demographic data including gender, age, and age distribution, are summarized in Table 2. Age frequency distribution revealed that most patients were aged 51–70, with only 4 cases being below the age of 50. The age distribution between male and female patients did not differ significantly (Mann-Whitney test *p* = 0.138, data not shown).

**TABLE 2:** Patient demographics.

Variable	Category	No. of cases		
		Total	Female	Male
Gender		53	25 (47.3%)	28 (52.7%)
Age	Minimum	31	31	35
	Median	62	59	63
	Maximum	80	71	80
	less than 50 years	4 (7.5%)	2	2
	51–60 years	20 (37.7%)	12	8
	61–70 years	24 (45.3%)	10	14
	more than 70 years	5 (9.4%)	1	4

### 3.2 Mutation spectrum in lung adenocarcinoma samples

**FIGURE 1:**
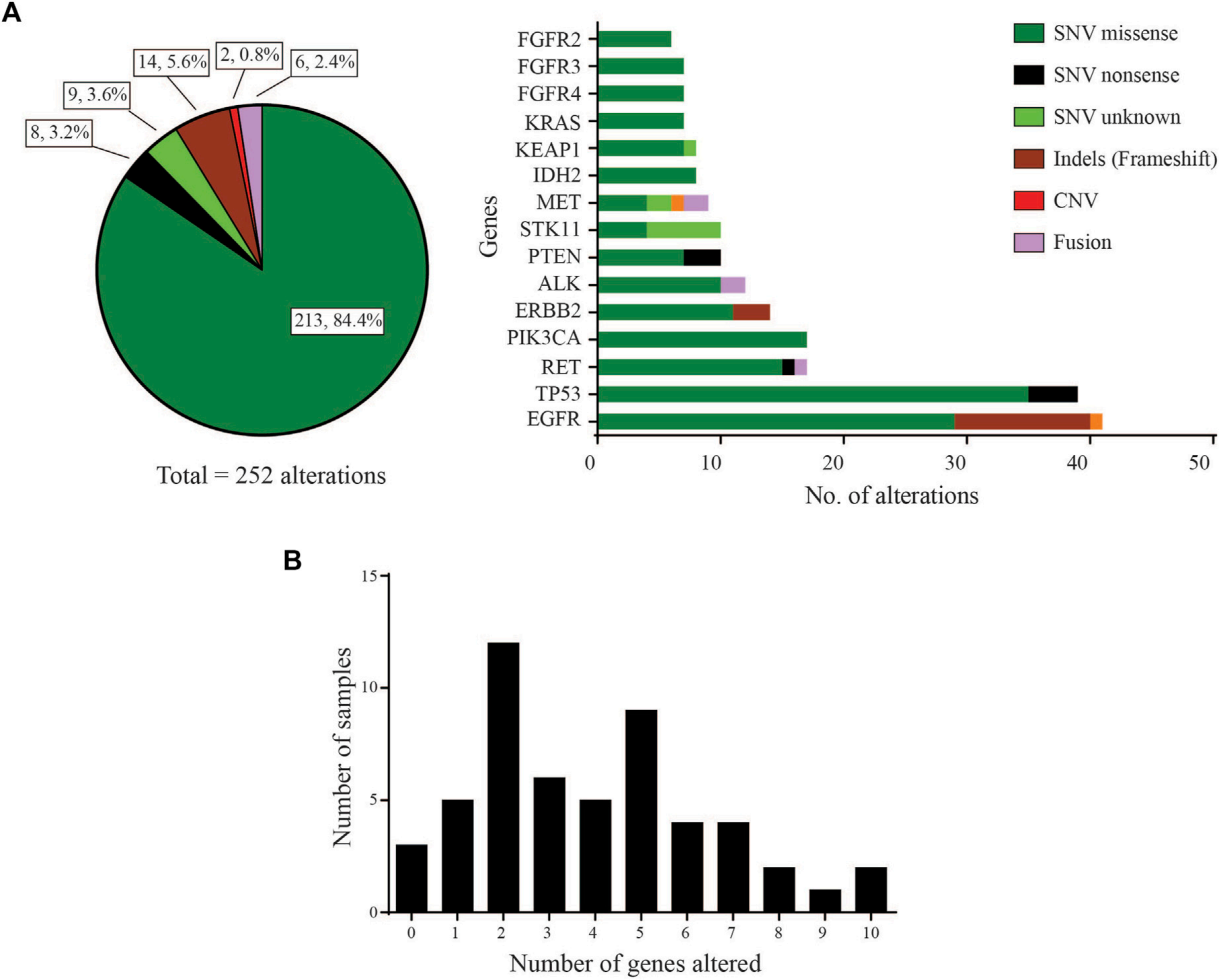
Gene distribution **(A)** Left: Pie-chart distribution of different types of genetic alterations detected. Right: Histogram of Gene-wise distribution of different types of genetic alterations in frequently mutated genes (>10% frequency distribution was used as cut-off for this figure). **(B)** Frequency of samples with number of distinct genes with at least one mutation altered in each sample.

Of the 53 LUAD cases analysed, 50 (94.3%) were found to carry at least one pathogenic or likely pathogenic variant using a variant allele frequency (VAF) cut-off of 2% (Supplementary data). These driver variants were detected in 31 genes out of the 46 cancer-relevant genes from the ODxET panel (See Supplementary methods). A total of 252 genetic alterations of different types were found in the hotspot regions of the 31 genes (Figure 1A). Some samples had multiple alterations in the same gene. The frequency distribution of distinct gene alterations with at least one mutation detected in them varied across the sample set, ranging from samples with no variants detected (*n* = 3, 5.7%), samples with only a single driver variant (*n* = 5, 9.4%), to samples with 10 genes alterations (*n* = 2, 3.8%) that included multiple alterations in oncogenes such as in *EGFR*, *PIK3CA*, *ERBB2*, *FGFR3* (Figure 1B).

**FIGURE 2:**
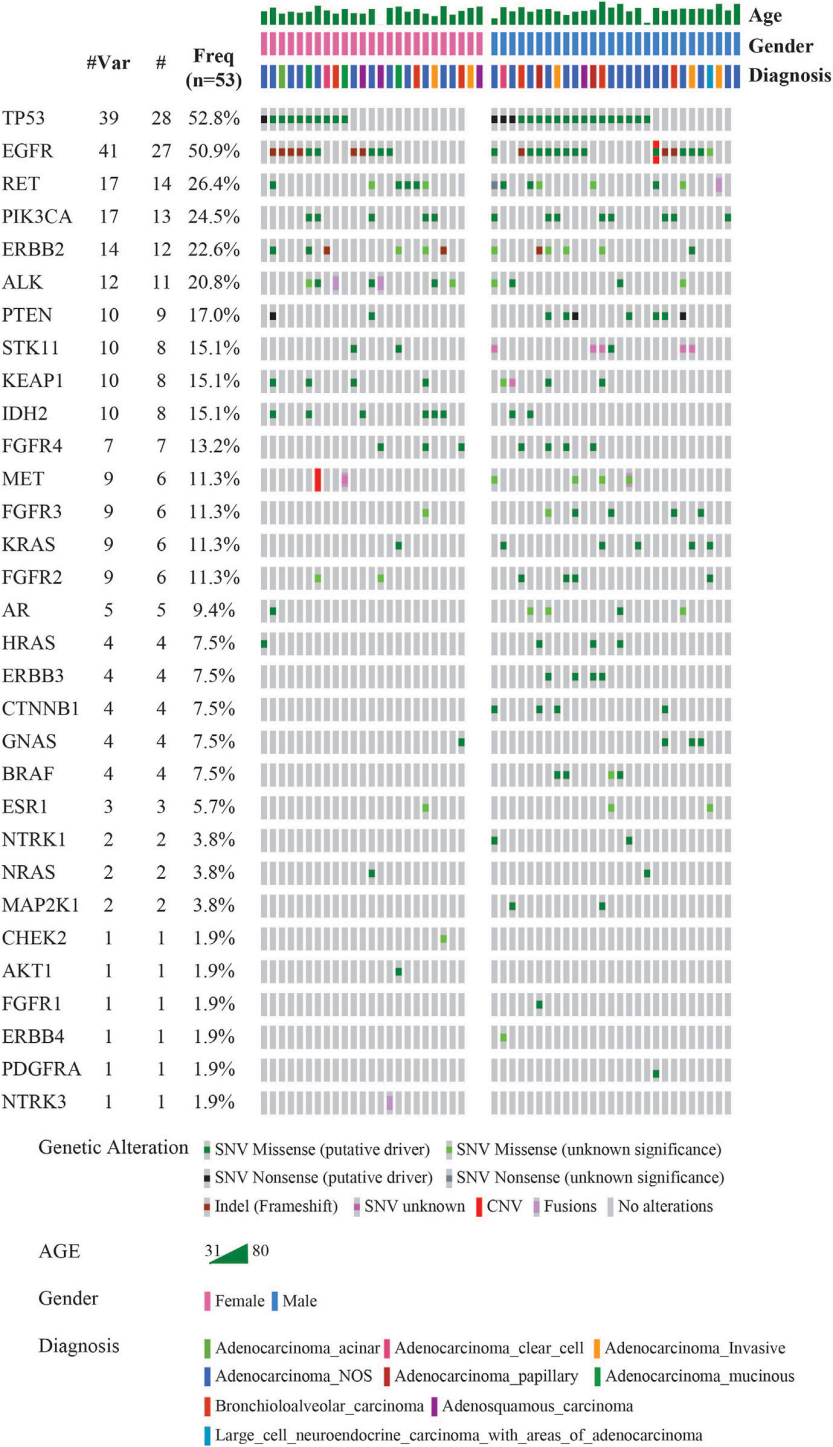
Mutational landscape of 53 LUAD visualized using OncoPrinter analysis in cBioPortal showing the gene alterations detected, frequencies of individual genes and other clinical-histopathological features (#-Total no. of cases with respective gene alteration observed with at least one mutation, #Mut-Total no. of mutations per gene across all cases, Freq-Frequency of gene alteration with at least one mutation (*n* = 53). (Altered in 50 (94.3%) of 53 samples using VAF cutoff of 2%).

The landscape of commonly mutated genes along with other clinical parameters was visualized using OncoPrinter analysis in the cBioPortal database (Figure 2). The most frequently altered genes comprised *TP53* (52.8%), and *EGFR* (50.9%), followed by *RET* (26.4%), *PIK3CA* (24.5%), *ERBB2* (22.6%), *ALK* (20.8%), *PTEN* (17.0%), *STK11*, *KEAP1* and *IDH2* (15.1%), *FGFR4* (13.2%), *MET*, *FGFR3*, *KRAS*, and *FGFR2* (11.3%).

**FIGURE 3:**
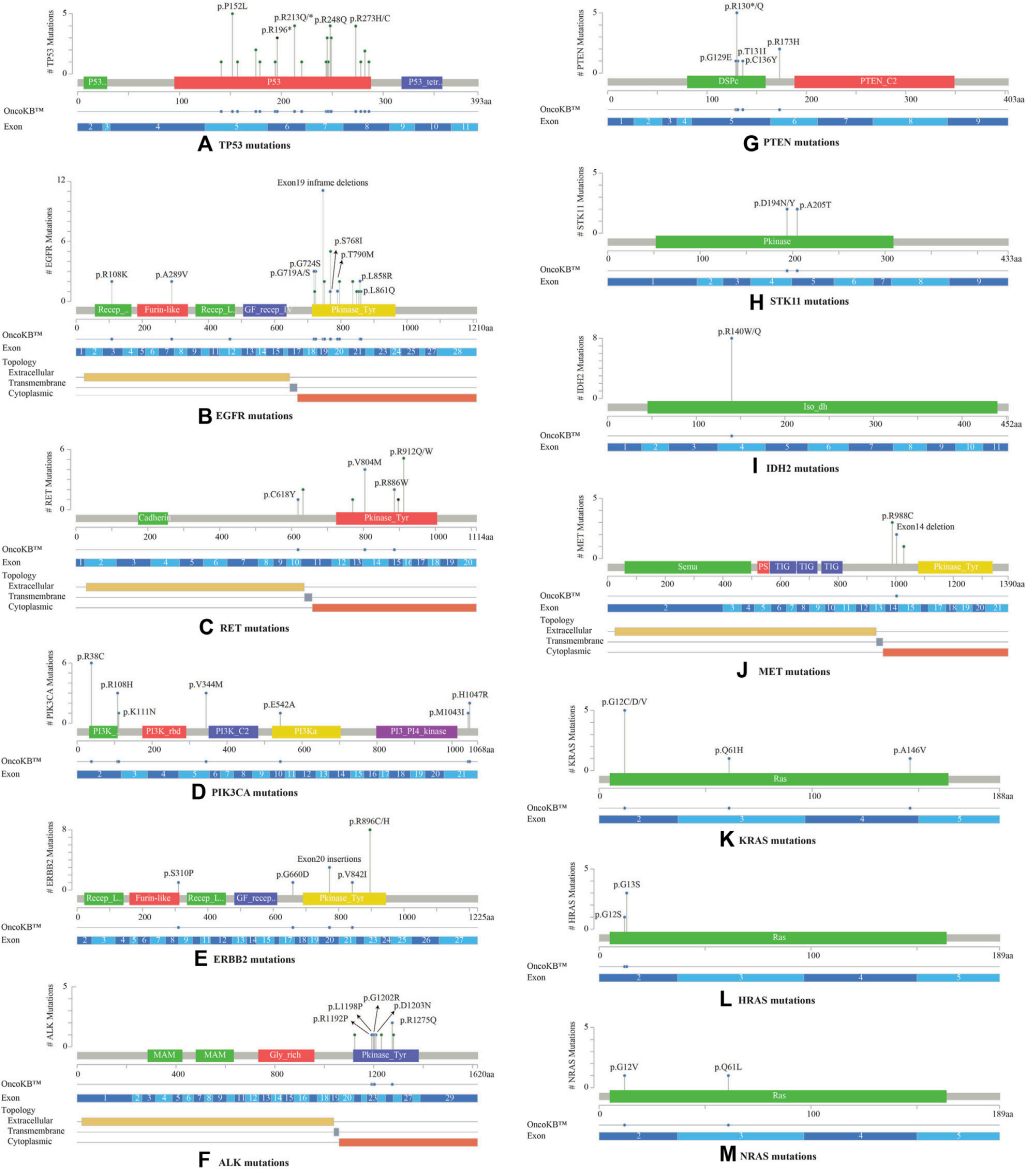
Lollipop plots visualizing the mutation spectra through the protein sequences of 13 genes with >10% frequency of observed mutations, using MutationMapper tab in the cBioPortal. Each lollipop represents a mutation identified in this study. The plot also highlights respective exon numbers and topological features (such as extracellular, transmembrane, and cytoplasmic). **(A)** TP53, **(B)** EGFR, **(C)** RET, **(D)** PIK3CA, **(E)** ERBB2, **(F)** ALK, **(G)** PTEN, **(H)** STK11, **(I)** IDH2, **(J)** MET, **(K)** KRAS, **(L)** HRAS, **(M)** NRAS.

Most *TP53* mutations were LOF missense mutations (*n* = 35), primarily occurring in exons 5 to 8 within the DNA binding domain (Figure 3A). *EGFR* mutations were detected in the cytoplasmic region from exons 18 to 21, located in the protein tyrosine kinase domains or the C-terminal phosphorylation domains. The most common *EGFR* mutation was Exon19 in-frame deletion (*n* = 9) (Figure 3B). *RET* variants were mostly GOF missense mutations that were predominantly located in the kinase domain, within the cytoplasmic region from exons 13 to 16. The most frequently observed mutation was *RET* V804M (Figure 3C). *RET* was the sole driver mutation in three cases: one case with *RET* C618Y mutation located in the cysteine-rich domain, another with *RET* V804M, and the third with a novel *RET-KIAA1468* fusion.

*PIK3CA* mutations were mostly observed in the catalytic subunit of PI3-kinase, including R38C in the p85 binding domain (*n* = 6), R108H in the adaptor-binding domain ABD (*n* = 3), V344M residue between the RAS binding domain and the C2 domain (*n* = 3), and H1047R in the kinase domain of the protein (*n* = 2) (Figure 3D). *PIK3CA* H1047R was the single driver mutation in one patient sample. Amplification in *ERBB2* (HER2 or ErbB2) was not observed in these 53 NSCLCs; instead activating oncogenic mutations were identified such as exon20 insertion p.Y772_A775dup (*n* = 3), V842I (*n* = 1), and G660D (*n* = 1). The most common *ERBB2* mutation observed was R896 C/H located in the kinase domain of the protein (*n* = 8) (Figure 3E).

*ALK* oncogenic mutations were primarily located in exons 23 to 26 of the gene, within the intracellular tyrosine kinase domain, with the most frequent ones being R1192P, L1198P, G1202R, D1203N, S1206Y and R1275Q (Figure 3F). Rearrangements of *ALK* were detected in 2 cases. *PTEN* LOF missense mutations were predominantly located in the phosphatase domain, with the truncating *PTEN* p.R130* being the most frequent mutation observed (*n* = 5) (Figure 3G). *STK11* mutations were identified at 3′-splicesite, 5′-splicesite, and tyrosine kinase domains, that included p.A205T and p.D194N/Y (Figure 3H). Other gene alterations with greater than 10% frequency included *IDH2* R140W/Q (*n* = 8) present in the catalytic domain of the protein (Figure 3I), *MET* exon 14 in-frame deletions (*n* = 2) (Figure 3J), *KRAS* exon 2 mutations (*n* = 6) located in the P-loop of the catalytic G-domain of the protein, HRAS exon 2 mutations (*n* = 4), NRAS G12V, Q61L (*n* = 2) (Figures 3K–M).

There were two cases of invasive adenocarcinoma treated with neoadjuvant chemotherapy (NACT) included in this study, each with distinct mutation profiles (SB00046727 and SB00046728). The first case exhibited a rare fusion mutation *ALK-PRKAR1A*, along with *FGFR4* V550M, *FGFR2* A648T, and *EGFR* V769M SNV mutations. The second case showed mutations in *IDH2* R140W and *EGFR* exon19 deletion. Furthermore, our study included two cases of locally relapsed NSCLCs. One case, classified as Bronchioloalveolar carcinoma with local recurrence (SB00036833), exhibited a mutation spectrum including *EGFR* exon19 deletion, *EGFR* A750P, *TP53* R249S, *FGFR4* V550M, and *FGFR2* S252L mutations. The second case, characterized as acinar adenocarcinoma with relapse within 6 months (SB00036841), presented mutations in *TP53* E286V, *TP53* R282W, and *EGFR* exon19 deletion.

### 3.3 Clinical correlations with genetic alterations

To check the correlation between the distribution of gene alterations with clinical features, an enrichment analysis was performed. No significant correlation was observed with age, tumor stage or subtype diagnosis of LUAD with mutations across any of the 46 genes.

**FIGURE 4:**
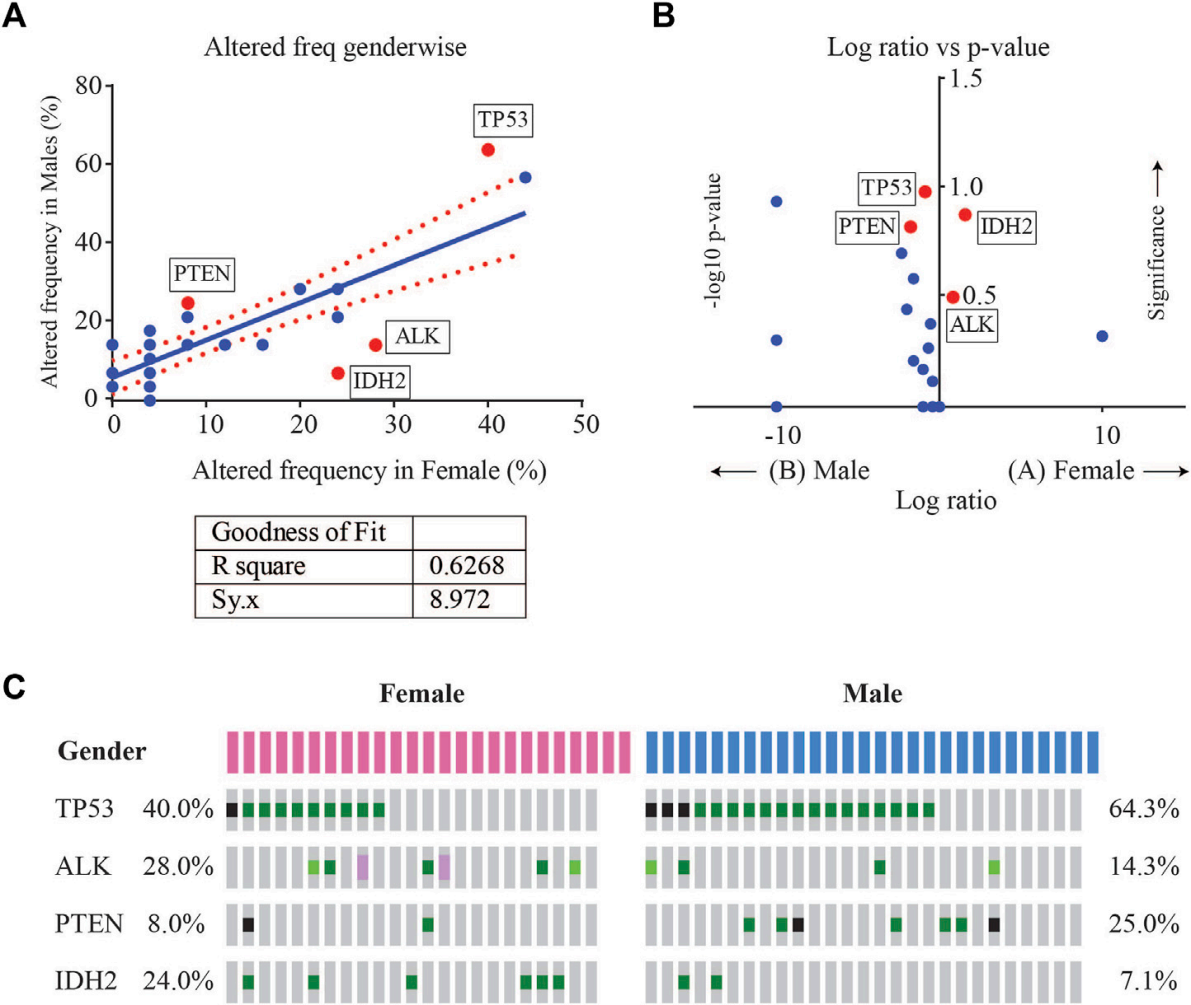
**(A)** Scatter plot of altered gene frequency enriched in male *versus* female patients. Linear regression analysis (R square = 0.6268) was performed using GraphPad **(B)** Volcano plot of log2 ratio of frequency occurrences in males and females and -log10 of *p*-value (derived from Fisher’s test) indicating the genes that were significantly enriched in male vs. female cases **(C)** Oncoprint visualization of gender-wise enriched genes in lung adenocarcinomas with gene frequencies.

Enrichment analysis of gene alterations by gender however, revealed that certain genes such as *ALK* and *IDH2* were more frequently enriched in females, while *TP53* and *PTEN* were more frequently enriched in males. *TP53* mutation frequency was 64.3% (18/28) in males as compared to 40% in females (10/25), with a *p*-value of 0.102. The mutation frequency of *IDH2* was found to be 7.1% (2/28) in males as compared to 24% in females (6/25), with a *p*-value of 0.129. Although not significant, these indicate a tendency towards gender-wise enrichment. These results were supported by scatter and volcano plots shown in Figure 4.

### 3.4 Actionable genetic alterations

**FIGURE 5:**
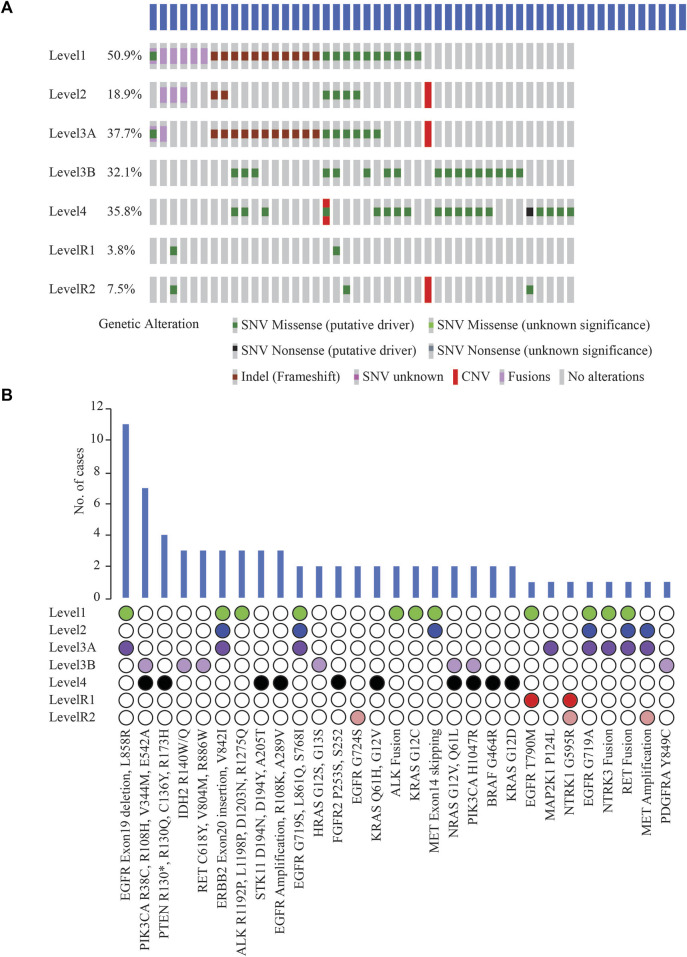
**(A)** List of clinically actionable alterations using 5% VAF filter and Oncoprinter analysis in cBioPortal, categorized into seven levels of evidence of their actionability, as classified by OncoKB (Level 1: FDA-approved biomarkers for NSCLC, Level 2: Strong clinical evidence supporting biomarker actionability to FDA-approved drug in NSCLC, Level 3A: Compelling clinical evidence supporting biomarker actionability in NSCLC, Level 3B: Standard care or investigational biomarker actionability to FDA-approved drug in another indication, Level 4: Preclinical evidence supporting biomarker actionability, Level R1: Biomarkers with resistance or futility to FDA-approved therapies in NSCLC, Level R2: Resistance biomarkers with compelling preclinical evidence of resistance to a drug) **(B)** Distribution of cases with clinically significant alterations, arranged based on the OncoKB level of clinical evidence of their actionability.

Potentially actionable somatic alterations were analysed using a higher, 5% VAF mutation sensitivity, that is more often used for clinical reporting. As defined by OncoKB Therapeutic Levels of Evidence V2 framework, these alterations were identified in 42 (79%) cases with at least one potentially actionable alteration eligible for adjuvant targeted therapy (Figure 5A). Of these 42 cases, 64.3% of patients (*n* = 27) had a Level 1 alteration, 2.4% (*n* = 1) had Level 2, 21.4% (*n* = 9) Level 3B, and 11.9% (*n* = 5) Level 4 as their highest level of actionability. More than one potentially actionable alteration was observed in 39.6% of patients (*n* = 21) that could help in selection of multiple lines of therapy. Biomarkers predictive of resistance or futility to FDA-approved therapies in NSCLC (R1) were also identified in 3.8% of cases (*n* = 2), and biomarkers with compelling preclinical evidence of resistance to a drug (R2) in 7.5% of cases (*n* = 4).

*EGFR* Exon19 in-frame deletions and L858R were the most frequent predictive biomarkers (*n* = 11, 20.8%), indicative of response to various EGFR TKIs (Figure 5B). Actionable *ERBB2* mutations (Exon20 insertion, G660D, V842I) were detected in 5.7% of cases (*n* = 3); these are predictive of response to Trastuzumab Deruxtecan, the first FDA-approved drug for HER2-mutant NSCLC. *EGFR* T790M detected in 1 case has been associated with acquired resistance to first- or second line- EGFR TKI therapies but predictive of a favourable response to Osimertinib. Oncogenic fusions in *ALK*, *RET*, *NTRK* family, *MET* exon14 alterations, all of which predict significant clinical benefit from targeted inhibitors of these kinases, were identified in 11.3% of cases (n = 6). Less common *ALK* oncogenic mutations (R1192P, L1198P, D1203N, S1206Y and R1275Q) were identified in 5.7% of cases (*n* = 3), associated with resistance to first and second-generation ALK inhibitors, and sensitivity to Lorlatinib. These resistance mutations, such as EGFR T790M and the less common ALK mutations, which are more frequently associated with disease progression on tyrosine kinase inhibitor (TKI) therapy, were not identified in the two cases of primary NSCLCs treated with NACT and the two cases of relapsed NSCLCs in our cohort. Previous reports have also indicated that these mutations have been identified in TKI-naive patients with NSCLC (Li et al., 2018); or as primary resistance mutations, leading to the lack of benefit from TKI treatment (Lin et al., 2017).

Other less common *EGFR* mutations observed, G719X, L861Q, S768I (*n* = 2) included as Level 1 are predictive of response to Afatinib. *KRAS* G12C (*n* = 2) is prognostic of poor survival and associated with responsiveness to Sotorasib and Adagrasib.

Among other genes not included yet in the NCCN guidelines for molecular testing in NSCLC, clinically informative alterations were detected in 9 cases (17.0%). These alterations included *PIK3CA* oncogenic mutations, namely, R38C, R108H, V344M, E542A, H1047R (*n* = 7); IDH2 R140W/Q (*n* = 3); *RET* oncogenic mutations such as *RET* C618Y, V804M, R886W (*n* = 3); *HRAS* G12S, G13S (*n* = 2); *NRAS* G12V, Q61L (*n* = 2); and *PDGFRA* Y849C (*n* = 1) (Figure 5B). Consistent with other studies, most oncogenic or likely oncogenic *PIK3CA* alterations were identified in tumors with a co-occurring higher OncoKB-level alteration (Jordan et al., 2017).

## 4 Discussion

The genetic heterogeneity and variations within and between different ethnic regions and countries highlights the need to explore the molecular characterization of lung cancers across diverse ethnic groups and populations (Park et al., 2018). Previous genomic studies in LUAD have focused on the mutational landscape more than their clinical and treatment implications (The Cancer Genome Atlas Research Network, 2014; Chen et al., 2020; Gillette et al., 2020). The ODxET panel used in this study is approved by FDA for use as a companion diagnostic to aid in selecting NSCLC and other solid cancers for treatment with approved targeted therapies. Our study determined the prevalence of molecular alterations in the commonly implicated genes in NSCLCs, followed by an in-depth analysis to evaluate the actionability of specific variants using OncoKB.

Of the 53 cases of primary non-metastatic LUAD, pathogenic or likely pathogenic alterations were detected in 50 (94.3%) cases in 31 of the 46 genes tested using a VAF cut-off of 2%. Absence of variants in 3 cases suggests that a different gene panel or whole exome sequencing maybe required for a small number of cases to identify variants not included in ODxET. No mutations were observed in 15 genes including *AKT2*, *AKT3*, *ARAF*, *CDK4*, *FLT3*, *IDH1*, *KIT*, *MAP2K2*, *NRG1*, *NTRK2*, *NUTM1*, *RAF1*, *ROS1*, *RSPO2*, and *RSPO3*. These genes have been reported to exhibit mutational incidence of <2% in NSCLCs (Imielinski et al., 2014; Dobashi et al., 2015; Anna F. Farago et al., 2018; Lui et al., 2018; Jonna et al., 2019; Azelby et al., 2021). Our previous study in 225 Indian LUAD patients had also reported a low mutational incidence of 0.4% in Indian NSCLCs for *ROS1* (Jain et al., 2019).

*TP53* and *EGFR* were the top 2 genes with the highest number of variants in this study. Although *TP53* mutation has been associated as a negative prognostic marker with poorer overall survival in advanced NSCLC, currently *TP53* deletions and oncogenic mutations are classified by OncoKB as prognostic level of evidence only for hematologic malignancies (Jiao et al., 2018). The frequency of *TP53* in this study (52.8%) was similar to TCGA data (47%) but higher than other targeted or whole exome studies, such as MSK-IMPACT (36%) and OncoSG (37%). *TP53* mutations were more enriched in males (Log ratio = −0.848, *p*-value = 0.1017) (Table 3).

**TABLE 3:** Comparison of NGS data of the most frequently altered genes (with greater than 10% frequency detected) in our study *versus* publicly available LUAD sequencing studies. In our study, the variant allele frequency (VAF) threshold used was 2%, while OncoSG used 8%, MSK-IMPACT used 10%, and TCGA’s VAF cutoff was not available (Collisson et al., 2014; Caso et al., 2020; Chen et al., 2020).

Article reference	TCGA (Nature, 2014)	MSK-IMPACT (JTO, 2020)	OncoSG (Nat Genet, 2020)	Present study
Year of study	2014	2020	2020	2022
Total samples	230	604	305	53
Type of study	WES of 230 lung adenocarcinoma tumor/normal pairs	Targeted sequencing of 604 lung adenocarcinoma tumor/normal pairs via MSK-IMPACT	WES of 305 East Asian lung adenocarcinomas with matched normals	Targeted NGS using ODxET panel
Most frequent mutations	TTN	KRAS	EGFR	TP53
Frequency of mutations (%)				
*TP53*	47	36	37	52.8
*EGFR*	17	31	49	50.9
*RET*	4	4	2.3	26.4
*PIK3CA*	9	6	4	24.5
*ERBB2*	5	6	7	22.6
*ALK*	8	4	4	20.8
*PTEN*	3	1.8	3	17.0
*STK11*	19	15	6	15.1
*KEAP1*	19	11	5	15.1
*IDH2*	1.7	0.5	0.3	15.1
*FGFR4*	4	3	4	13.2
*MET*	11	7	2.6	11.3
*FGFR3*	1.7	0.7	1	11.3
*KRAS*	36	38	13	11.3
*FGFR2*	4	2	1	11.3

The most common *EGFR* mutations were short in-frame deletions of exon19 (*n* = 9, 17.0%). Other less common *EGFR* mutations (9.4%) included G719X (*n* = 3) in exon 18, S768I in exon 20 (*n* = 1), and L861Q in exon 21 (*n* = 1). This frequency of uncommon *EGFR* mutations was similar to other studies (11.9%–13.4%) (Tu et al., 2017; Kaler et al., 2022). An analysis examining the mutual exclusivity between *EGFR* and *KRAS* mutations indicated a tendency towards mutual exclusivity, although it did not reach statistical significance (log odd ratio = −1.396, *p* = 0.395, q = 1). This finding is consistent with previous studies in Western populations, where *EGFR*, *KRAS*, and *ALK* genetic alterations were reported to be predominantly mutually exclusive in NSCLC (Gainor et al., 2013). The frequency of *EGFR* mutations (50.9%) was notably higher in the present study than other Western studies (17% in TCGA and 31% in MSK-IMPACT), but similar to Singapore patients (49%). Mutations in some genes such as *KRAS*, for which a new drug was approved in 2022, were present at a lower frequency in our study as well as Singapore patients (10.9%–13%) as compared to other Western studies (36%–38%) (Table 3).

A rare *RET- KIAA1468* fusion was detected in a patient with mucinous LUAD. This fusion was previously reported in other mucinous LUAD cases, often occurring mutually exclusively with *KRAS* mutations (Nakaoku et al., 2014; Jiang et al., 2020). The most frequent *ERBB2* mutation observed was R896C/H (15.1%). The biological significance of *ERBB2* R896 C/H is not well established but it has been found to increase the *in vitro* kinase activity of HER2 and enhance HER2, EGFR, and PLCγ phosphorylation in MCF10A cells (Bose et al., 2013). **A** rare genetic alteration of *ALK-PRKAR1A* was identified, previously found in an NSCLC patient who responded to crizotinib (Du et al., 2021). A novel ALK fusion was detected using 5’/3’ imbalance strategy developed for specific and sensitive detection of ALK fusions (Tong et al., 2018).

The OncoKB database offers clinicians and researchers a comprehensive and curated repository of cancer-related genetic variants and their therapeutic implications, facilitating well-informed decisions regarding personalized cancer treatments (Chakravarty et al., 2017). The FDA has acknowledged it as a reliable source of information for tumor profiling tests (U.S. Food and Drug Administration, 2021).

At least one clinically significant alteration (Level 1–4) as annotated by OncoKB was detected in 42 (79%) cases using the 5% VAF threshold, involving 18 actionable genes. Among these, 27 (50.9%) patients had a Level 1 alteration, that includes the actionable biomarkers recommended by the NCCN Guidelines for molecular testing in NSCLC. Targetable *EGFR* alterations (Exon19 in-frame deletions and L858R) were the most frequent biomarker (*n* = 11, 20.8%) predictive of response to several EGFR TKIs. Another study had reported a similar higher frequency of targetable *EGFR* alterations in LUAD (Caso et al., 2020). These findings suggest the likelihood of these patients responding positively to paired biomarker-associated targeted therapies approaches.

Multiple potentially actionable alterations were found in 21 (39.6%) patients. For example, a case of lung invasive adenocarcinoma exhibited two different alterations in the *EGFR* gene, a Level 1 (Exon19 deletion) and a Level R1 alteration (T790M). The Exon19 deletion in *EGFR* is associated with response to drugs such as Afatinib, Dacomitinib, Erlotinib, Gefitinib, and Osimertinib. The *EGFR* T790M alteration on the other hand, is associated with resistance to drugs such as Erlotinib, Gefitinib, and Afatinib, while predicting a positive response to Osimertinib. Therefore, integrating information from both mutations underscores the high utility of genetic data for precision medicine.

Among the clinically predictive biomarkers recommended for testing as per the NCCN clinical guidelines version 2.2023 in NSCLC, no alterations or rearrangements were detected in *NTRK1*, *NTRK2*, or *ROS1* in these samples. In our study, 42 (80%) cases had potentially actionable levels as per OncoKB database as compared to only 27 (52.7%) as per current NCCN guidelines for molecular testing. This is because OncoKB may integrate and suggest refinement or expansion to existing practices or evidence levels based on FDA-approvals for drugs in other indications for the same mutations, peer-reviewed scientific literature, or clinical trials (Chakravarty et al., 2017). Among other genes not listed in NCCN guidelines for molecular testing for NSCLC, clinically informative alterations were identified in 9 (17.0%) cases, currently recognized as standard of care or investigational biomarker actionability to FDA-approved drug in another indication, but not yet approved for NSCLC. These alterations, including oncogenic mutations in *PIK3CA* and *IDH2*, have already been categorized as Level 1 alterations in other cancer types, but are still emerging as potential therapeutic targets in NSCLC (Scheffler et al., 2014; Rodriguez et al., 2020). These alterations have the potential to be expanded to NSCLC through dedicated clinical trials and accumulation of real-world evidence, based on promising outcomes in NSCLC patients with these alterations.

In summary, the results from this study identified pathogenic or likely pathogenic alterations in 31 genes, of which 18 are known to be clinically actionable. The use of a smaller NGS panel could improve clinical accessibility and cost-effectiveness, especially in a low resource country like India. In this context, our study demonstrates the utility of such targeted NGS panels for identifying the most common alterations and using their biological and clinical implications for personalizing medicine for Indian LUAD patients, a cohort that is underrepresented in public databases.

## Data Availability

The data presented in this study are deposited in the European Variant Archive repository. The accession number for the project is PRJEB69688 and for the analyses is ERZ22146218.
