# Supplementary material for: The landscape of actionable genomic alterations in lung adenocarcinomas in India
Source: Front Genet. 2023 Dec 13;14:1256756. doi: 10.3389/fgene.2023.1256756 (PMC10754624; doi:10.3389/fgene.2023.1256756)
Supplement: Supplementary file 1 [file Table2.docx]

**Supplementary Methods**

**Next generation sequencing**

The ODxET^TM^ is a 46 gene panel NGS assay that utilizes amplicon-based unique molecular identifiers (UMIs). The ODxET panel analyses somatic variants frequently observed in cancer samples implicated with relevant cancer phenotype, and certain less frequently occurring variants that are functionally relevant in tumorigenesis as published in literature reports. These variants include 42 DNA hotspots, 11 CNVs, 16 inter-genetic fusions, and 3 intra-genetic fusions.

The DNA panel is designed to identify hotspot mutations in the following 42 cancer-relevant genes: *AKT1*, *AKT2*, *AKT3*, *ALK*, *AR*, *ARAF*, *BRAF*, *CDK4*, *CHEK2*, *CTNNB1*, *EGFR*, *ERBB2*, *ERBB3*, *ERBB4*, *ESR1*, *FGFR1*, *FGFR2*, *FGFR3*, *FGFR4*, *FLT3*, *GNAS*, *HRAS*, *IDH1*, *IDH2*, *KEAP1*, *KIT*, *KRAS*, *MAP2K1*, *MAP2K2*, *MET*, *NRAS*, *NTRK1*, *NTRK2*, *NTRK3*, *PDGFRA*, *PIK3CA*, *PTEN*, *RAF1*, *RET*, *ROS1*, *STK11* and *TP53*. The RNA panel can identify rearrangements in 11 genes namely *AR*, *EGFR*, *ERBB2*, *ERBB3*, *FGFR1*, *FGFR2*, *FGFR3*, *KRAS*, *MET*, *PIK3CA* and *PTEN*. The ODxET^TM^ test uses FusionSync^TM^ (Exon-tiling imbalance approach) that allows for the detection of previously unknown gene fusions, apart from a targeted panel designed for known gene fusions. The fusion gene panel includes 11 inter-genetic fusion genes: *ALK*, *BRAF*, *ESR1*, *FGFR1*, *FGFR2*, *FGFR3*, *MET*, *NRG1*, *NTRK1*, *NTRK2*, *NTRK3*, *NUTM1*, *RET*, *ROS1*, *RSPO2*, *RSPO3* and 3 intra-genetic fusion genes namely *AR*, *EGFR*, and *MET*.

DNA and RNA were simultaneously extracted using the MagMax^TM^ FFPE DNA/RNA Ultra kit (ThermoFisher Scientific, Waltham, MA, USA) according to manufacturer’s protocol. The optimal input of solid tumor (FFPE DNA or RNA) is 0.67 ng/µL for Genexus- ODxET, with a verified nucleic acid concentration range of 0.33-1 ng/µL. The ODxET ^TM^ protocol (Thermo Fisher Scientific, 2020) was used for the library preparation, including reverse transcription, amplification, and ligation steps.

**Variant calling and data analysis**

Raw data processing, alignment, and variant calling were performed by ThermoFisher Scientific (TFS) using their Torrent Suite software, and variants were annotated with the Torrent Variant Caller plug-in, followed by downstream analyses by Ion Reporter software with the workflow “Oncomine Focus Assay” selected and filter chain “Oncomine Variant Annotator” applied. The hg19 was used as the reference genome to annotate the variants, and assignment of identifiers consistent with the publicly accessible databases for clinically relevant mutations, such as COSMIC, dbSNP and ClinVar, and arbitrary identifiers assigned by the software for future interpretation and troubleshooting as and when such variants are annotated in public data sources.

The quality check (QC) metrics comprised multiple parameters and tests, such as purity and DNA/ RNA concentrations; run QC (key signal, percent loading, raw read accuracy), templating QC (average reads per lane, base call accuracy, mean AQ20 read length), NGS QC of DNA (concentration > 9 ng/µl; mapped reads >= 400,000, Median of the Absolute values of all Pairwise differences (MAPD) <= 0.5, uniformity of base coverage >= 88%, mean read length >=35 , total end-to-end mapped reads >= 50%), NGS QC for RNA (total mapped reads >= 20,000, mean read length >= 30, RNA expression controls >= 5). The final QC score for each sample was calculated based on the above metrics and assigned “good”, “questionable”, or “poor”. Samples labelled as ‘poor’, that failed QC metrics in more than 1 QC metric, were excluded from this study. Samples that passed two or more DNA or RNA QC metrics (‘questionable’ and ‘good’) were included in the analysis.
